# Macular hole formation following intravitreal injection of ranibizumab for branch retinal vein occlusion: a case report

**DOI:** 10.1186/s13104-015-1324-4

**Published:** 2015-08-19

**Authors:** Daisuke Muramatsu, Ryosuke Mitsuhashi, Takuya Iwasaki, Hiroshi Goto, Masahiro Miura

**Affiliations:** Tokyo Medical University, Ibaraki Medical Center, 3-20-1 Chuo, Ami-machi, Inashiki-gun, Ibaraki 300-0395 Japan; Tokyo Medical University, 6-7-1 Nishishinjuku, Shinjuku-ku, Tokyo, 160-0023 Japan

**Keywords:** Branch retinal vein occlusion, Macular hole, Ranibizumab, Macular edema, Complication

## Abstract

**Background:**

Macular hole formation after anti-vascular endothelial growth factor therapy is a rare complication. We report macular hole formation after intravitreal ranibizumab injection for branch retinal vein occlusion.

**Case presentation:**

A 63-year-old Asian male was treated with intravitreal ranibizumab injection for chronic macular edema with branch retinal vein occlusion in his right eye. Before treatment, best-corrected visual acuity in his right eye was 20/200. Nine days after injection, a full thickness macular hole developed with reduction of macular edema. After pars plana vitrectomy combined with cataract surgery, the macular hole was successfully closed, and the best-corrected visual acuity in his right eye improved to 20/40.

**Conclusion:**

The possibility of an infrequent complication like macular hole should be considered for intravitreal ranibizumab for macular edema with branch retinal vein occlusion.

## Background

Retinal vein occlusion (RVO), including branch retinal vein occlusion (BRVO), is a major retinal vascular disease. Macular edema is the most common cause of visual impairment in eyes with RVO. There are several treatment options for macular edema in RVO, including laser photocoagulation [1], intravitreal steroid treatment [2], and vitrectomy [3]. Recently, various anti-vascular endothelial growth factor (VEGF) therapies including ranibizumab [4] (Lucentis^®^; Genentech, South San Francisco, CA, USA), aflibercept [5] (Eylea^®^; Regeneron, Tarrytown, PA, USA and Bayer HealthCare, Berlin, Germany), and bevacizumab [6] (Avastin^®^; Genentech) are widely used for the treatment of macular edema in RVO. Anti-VEGF therapy has shown favorable results for RVO [4–6], although adverse complications have been reported with this treatment [4]. We report a patient who developed a macular hole (MH) after ranibizumab injection for chronic BRVO, with closure of this macular hole after vitrectomy.

## Case presentation

**Fig. 1:**
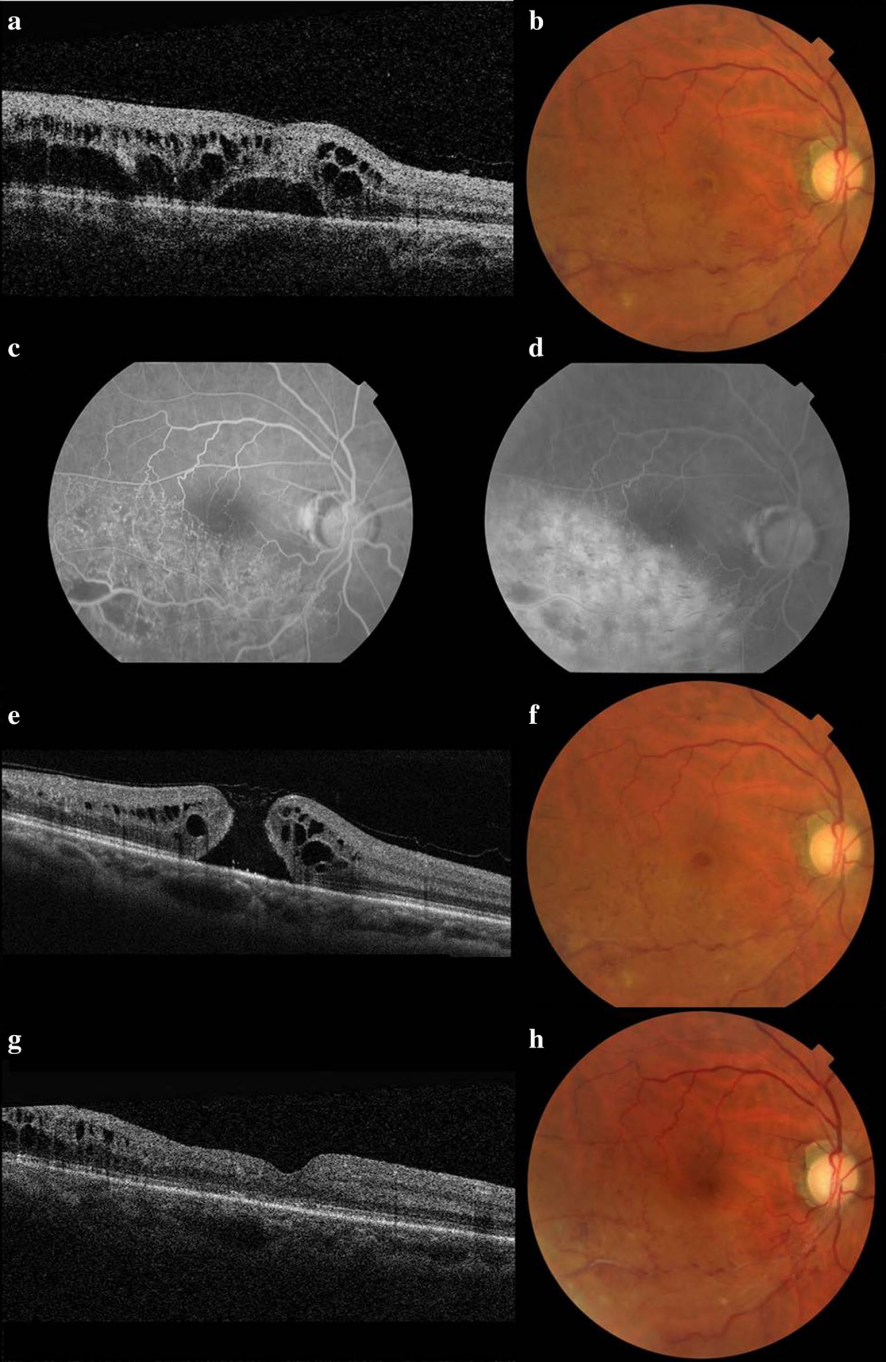
B-scan optical coherence tomography (OCT) and color fundus photography before and after treatment. **a**, **b** Findings in the right eye before intravitreal administration of ranibizumab for chronic branch retinal vein occlusion. Serous retinal detachment and macular edema were located at the outer retina. **c**, **d** Fluorescein angiography revealed a capillary nonperfusion area and dye leakage in the late phase. **e**, **f** Nine days after injection of ranibizumab, OCT and color fundus photography showed formation of a full thickness macular hole and decreasing intraretinal edema. **g**, **h** Five months after surgery, the macular hole was closed and macular edema decreased

A 63-year-old Asian male, diagnosed with BRVO with refractory macular edema, was referred from his home doctor. His vision in his right eye had been slowly decreasing without any treatment for the previous 5 years. The best-corrected visual acuity (BCVA) in his right eye was 20/200 and conventional fundus examination showed retinal hemorrhage followed by chronic BRVO in his right eye. He had a past history of hypertension, but no hyperlipidemia or diabetes mellitus. Optical coherence tomography (OCT) revealed serous retinal detachment and intraretinal edema that was located at the outer retina (Fig. 1a). Central retinal thickness (CRT) from the OCT B-scan image was 542 µm. Using fluorescein angiography imaging, a small capillary nonperfusion area with collateral vessel formation was detected in the early phase, and dye leakage in the area of the vein occlusion was detected in the late phase (Fig. 1c, d). Slit lamp biomicroscopy revealed that the posterior vitreous cortex was attached on the macula. After obtaining informed consent, the patient was administered 0.5 mg ranibizumab intravitreally using a 32 gauge needle. Nine days after injection, BCVA in his right eye improved to 20/100. OCT showed the formation of a full thickness MH and decreasing intraretinal edema (Fig. 1e). Twenty-two days after injection, BCVA further recovered to 20/50, but the MH was still open. The patient underwent 25 gauge pars plana vitrectomy, combined with cataract surgery and intraocular lens implantation. Internal limiting membrane peeling, photocoagulation to the nonperfusion area, and gas tamponade using 20 % sulfur hexafluoride were successfully performed. One day after surgery, OCT confirmed successful closure of the MH. Five months after surgery, BCVA recovered to 20/40 and CRT decreased to 272 μm (Fig. 1g).

## Discussion

There have been several reports of MH formation after intravitreal anti-VEGF therapy [7–12]. They included myopic choroidal neovascularization [7], age-related macular degeneration (AMD) [8–10], polypoidal choroidal vasculopathy [11], and hemicentral retinal vein occlusion [12]. However, to the best of our knowledge, this is a first report of MH formation after anti-VEGF therapy for BRVO.

The mechanism of MH formation after choroidal neovascularization (CNV) treatment may have involved the rapid volume reduction of CNV after anti-VEGF therapy [7, 8]. However, Grigoropoulos et al. [10] described the cause of MH formation as a force not only to the retinal pigment epithelium (RPE), but also to the retinal surface. Querques et al. [9] reported increasing vitreous macular traction after ranibizumab injection, with formation of a stage 2 MH. In AMD treatment with anti-VEGF therapy, the responsible factors for MH formation were assumed to exist at the RPE, retinal surface, and vitreous [7].

Nagpal et al. reported MH formation after bevacizumab injection to a hemi-central retinal vein occlusion patient. In this patient, rapid posterior vitreous detachment (PVD) after injection was a possible causative factor for the development of the MH [12]. In our case, the PVD itself might not have been a causative factor for MH formation, because PVD was induced with active aspiration during surgery. However, as in former reports, there was a possibility of vitreous macular traction.

The expression of transforming growth factor (TGF)-β2, which is known to cause fibrosis, was reported after anti-VEGF therapy [13, 14]. In proliferative diabetic retinopathy, acceleration of pathologic fibrosis by anti-VEGF therapy was also reported [15]. In our case, acute regression of macular edema and increased fibrosis might have triggered a mechanical force to the damaged retina, and might have been a possible cause of MH formation.

## Conclusions

Anti-VEGF therapy is a powerful and standard modality for management of macular edema following BRVO. However, the possibility of infrequent complications like MH should be considered after intravitreal injection of ranibizumab.

## Consent

Written informed consent was obtained from the patient for publication of this Case Report and any accompanying images. A copy of the written consent is available for review by the Editor-in-Chief of this journal.

## Abbreviations

RVO: retinal vein occlusion
BRVO: branch retinal vein occlusion
VEGF: vascular endothelial growth factor
MH: macular hole
BCVA: best-corrected visual acuity
OCT: optical coherence tomography
CRT: central retinal thickness
AMD: age-related macular degeneration
CNV: choroidal neovascularization
RPE: retinal pigment epithelium
PVD: posterior vitreous detachment
TGF: transforming growth factor
